# Evaluation of the efficacy as well as prognosis of targeted therapy for advanced non-small cell lung cancer patients with different expression of miR-183 family in body fluids

**DOI:** 10.3389/fmed.2025.1738202

**Published:** 2026-01-12

**Authors:** Min Chen, Yahui Shen

**Affiliations:** Department of Respiratory and Critical Care Medicine, The Affiliated Taizhou People’s Hospital of Nanjing Medical University, Taizhou School of Clinical Medicine, Nanjing Medical University, Taizhou, Jiangsu, China

**Keywords:** EGFR-TKI, miR-183, non-small cell lung cancer, prognosis, respiratory

## Abstract

**Aim:**

To explore the relationship between the expression of miR-183 family in body fluids and the treatment effect and prognosis of advanced non-small cell lung cancer (NSCLC) patients received epidermal growth factor receptor tyrosine kinase inhibitor (EGFR-TKI).

**Methods:**

One hundred and fifty advanced NSCLC patients were selected as the study subjects, all of whom received EGFR-TKI osimertinib. The efficacy of all NSCLC patients was evaluated after 2 courses of treatment, and the patients were allocated into effective group (*n* = 40) and ineffective group (*n* = 110). Real-time fluorescence quantitative PCR detected the relative expression of serum miR-183 in both groups. The EGFR-TKI efficacy of NSCLC patients with different clinical characteristics along with the survival rate of patients with different serum miR-183 relative expression levels before treatment was compared. Multivariate Cox regression model was implemented to analyze the factors affecting survival of NSCLC patients after EGFR-TKI treatment.

**Results:**

After treatment, serum miR-183 expression in effective group was declined relative to before treatment and ineffective group, and serum miR-183 expression in ineffective group was elevated compared to before treatment (all *P* < 0.05). The EGFR-TKI efficacy of NSCLC patients with no smoking history and miR-183 relative expression level <1.77 before treatment was better than that of NSCLC patients with smoking history and miR-183 relative expression level ≥1.77 before treatment (all *P* < 0.05). The 2-years survival rate in patients with miR-183 relative expression level <1.77 before treatment was elevated compared to that in patients with miR-183 relative expression level ≥1.77 before treatment (all *P* < 0.05). The relative expression of miR-183 before treatment was an independent influencing factor for survival of NSCLC patients after EGFR-TKI treatment (both *P* < 0.05).

**Conclusion:**

The low serum miR-183 expression before treatment is closely linked to the efficacy along with prognosis of advanced NSCLC patients received EGFR-TKI therapy. Monitoring the level of serum miR-183 may be helpful to evaluate the prognosis of NSCLC patients.

## 1 Introduction

Non-small cell lung cancer (NSCLC) occupies over 85% of all kinds of lung cancer (1). Its onset is insidious and no obvious symptoms can be discovered in the early stage, so most patients received diagnosis in the advanced clinical stage and miss the opportunity for surgical treatment (2). In recent years, molecularly targeted agents, epidermal growth factor receptor tyrosine kinase receptor inhibitors (EGFR-TKI), including osimertinib and erlotinib, have exhibited good antitumor effects in both NSCLC (3–5). These drugs inhibit intracellular tyrosine kinase phosphorylation by competing with ATP for intracellular tyrosine kinase sites, block EGFR signaling, and inhibit downstream effects of the pathway, including inhibiting cell proliferation, survival, and angiogenesis (6, 7).

At present, the therapeutic effect of NSCLC patients is usually evaluated by reexamination of MRI or CT, and some common serum tumor markers can also be used as auxiliary indicators to evaluate the therapeutic effect of NSCLC patients (8). MicroRNAs (miRNAs) belong to a group of 21–25 nucleotides non-coding RNAs that combines with 3’-untranslated region (3’-UTR) of mRNAs, bringing out transcript degradation or translational inhibition (9). MiRNAs participate in multiple cellular processes, containing proliferation, differentiation along with apoptosis (10). MiRNA deregulation belongs to a hallmark of many human cancers, and they can be divided into oncogenic miRNAs or tumor repressive miRNAs (11, 12). For instance, MiR-1299a functions as an onco-miRNA in NSCLC and promote NSCLC growth via down-regulating the expression of SOX6 (13). MiR-621 represses the progression of NSCLC by modulating SIX4 expression (14). Thus, miRNAs can be emerged to be potential biomarkers in lung cancer research. Among candidate miRNAs, miR-183 stands out for NSCLC: it is overexpressed in tumors and circulation and associates with unfavorable prognosis (15–17). Mechanistically, miR-183 directly represses PTEN and FOXO1-critical negative regulators of EGFR/PI3K/AKT signaling, providing *a priori* biological rationale to evaluate its relationship with EGFR-TKI efficacy (18–20).

Consistent with this rationale, miR-183 is measurably elevated in the circulation of patients with lung cancer and has been proposed as a minimally invasive biomarker, motivating our focus on serum miR-183 in the present study (16, 17, 21). Nevertheless, there are few researches on the relation between serum miR-183 level and the efficacy and prognosis of EGFR-TKI treatment in advanced NSCLC patients. Based on this, this paper investigated the relation between serum miR-183 level and the therapeutic effect and prognosis of NSCLC patients received EGFR-TKI therapy.

## 2 Data and methods

### 2.1 Clinical data

One hundred and fifty advanced NSCLC patients admitted to our hospital from January 2018 to December 2023 were chosen to be the study objects. There were 70 men together with 80 women, aged from 45 to 80 years, the mean age was (65.58 ± 10.42) years. There were 100 patients having smoking history and 50 cases without smoking history. All patients were diagnosed with adenocarcinoma.

Inclusion criteria: (1) Patients met the diagnostic criteria for NSCLC, and were confirmed by cytology and histology. (2) All patients were primary tumor-regional lymph node-distant metastasis stage III–IV. (3) There were no contraindications for the drugs used in this study. (4) Complete clinical examination data. (5) All patients and their family members gave informed consent to this study and voluntarily participated in it.

Exclusion criteria: (1) Patients with severe cardiovascular disease. (2) Patients with other malignant tumors and severe liver and kidney dysfunction. (3) Lost visitors. (4) Patients with unclear pathological diagnosis.

In total, 150 patients were included for analysis (Effective *n* = 40; Ineffective *n* = 110). To enhance clarity, the consolidated sample accounting is summarized in Table 1, and complete eligibility criteria are presented in Supplementary Table 1.

**TABLE 1:** Baseline characteristics and consolidated sample accounting (*N* = 150).

Category	Level	Total *n*	Total%	Effective *n*	Effective%	Ineffective *n*	Ineffective%	χ^2^	*P*-value
Age	<65 years	91	60.7	25	62.5	66	60.0	0.077	0.7816
	≥65 years	59	39.3	15	37.5	44	40.0		
Gender	Male	70	46.7	17	42.5	53	48.2	0.38	0.5373
	Female	80	53.3	23	57.5	57	51.8		
Smoking history	Yes	97	64.7	12	30.0	85	77.3	28.689	<0.0001
	No	53	35.3	28	70.0	25	22.7		
Baseline serum miR-183	<1.77	63	42.0	28	70.0	35	31.8	17.555	<0.0001
	≥1.77	87	58.0	12	30.0	75	68.2		

This study was reviewed and approved by the Ethics Review Committee of Taizhou People’s Hospital of Jiangsu Province (Approval No. KY201804601; initial review, quick review; date of review: January 18, 2018; valid January 2018–December 2023). All participants (or their legal guardians) provided written informed consent prior to inclusion.

### 2.2 Methods

#### 2.2.1 Treatment and follow-up methods

All patients with NSCLC were given EGFR-TKI osimertinib mesylate tablet (AstraZeneca, UK), 80 mg each time, once a day (21 days for 1 course) until the tumor became intolerable or progressed, and conventional local radiotherapy was given. Specific radiotherapy plan: 6MV-X, 4 field irradiation were selected for radiotherapy, 5 times/week, 2–3 Gy/times, a total of 4 weeks. Head and chest CT, MRI-abdominal B-ultrasound, and whole body bone SPECT/CT scans were performed monthly to evaluate the extent and size of metastases.

All 150 NSCLC patients received followed up from the time they received EGFR-TKI treatment until December 31, 2024, or until death.

#### 2.2.2 Efficacy evaluation

The efficacy of osimertinib in NSCLC patients was evaluated after 2 courses of treatment, based on head and chest CT, MRI, chest and abdomen B-ultrasound scanning, and whole-body bone SPECT/CT scanning, and the efficacy was assessed on the basis of RECIST 1.1 solid tumor efficacy evaluation criteria. Among them, full remission represented that all tumor lesions disappear after treatment and maintain for more than 1 month. Partial remission referred to the reduction of the maximum total diameter of tumor lesions by >30% after treatment, and it was maintained for more than 1 month. Stable disease meant that the diameter of tumor lesions decreases slightly after treatment, but did not reach the level of partial remission, or the maximum diameter of tumor lesions increased, but did not exceed 20%. Disease progression referred to an increase of more than 20% in the total maximum diameter of tumor lesions after treatment, or the emergence of new lesions. Total response rate = (total response + partial response)/total response × 100%. NSCLC patients were separated into effective group and ineffective group following the treatment effect, patients with complete remission or partial remission were contained in the effective group, and patients with stable or progressive disease were contained in the ineffective group.

#### 2.2.3 Serum sample collection

Before treatment and after 2 courses of treatment, 3–4 mL of fasting elbow venous blood was gathered from all patients in the morning. Followed by centrifugation, and serum was obtained, which was packaged and sealed in a refrigerator at −80 °C for future examination.

#### 2.2.4 Detection of the serum miR-183 expression by real-time fluorescence quantitative PCR

Serum samples were thawed and frozen, and total RNA was taken to extract by TRIzol reagent (Thermo Fisher, USA). The DNA was taken to reversely transcribe into cDNA according to the TaqMan^®^ MicroRNA Reverse Transcription Kit (Thermo Fisher, USA). PCR amplification was performed by preparing a reaction system with appropriate amount of cDNA as template and referring to the instruction manual of TaqMan^®^ MicroRNA Assay (Thermo Fisher, USA). U6 was used as the internal reference gene. The primer sequences of miR-183 and U6 were as indicated. MiR-183 F: 5′-AGUGAAUUCUACCAGUGCCAUC-3′ R: 5′-AGG AAUUCUACCAGUGCCAUC-3′, U6 F: ′-CTCGCT TCGGCAGCACA-3′, and R: 5′-CGCTTCACGAATTTGCGT-′. The relative miRNA-183 expression was taken to calculate by 2^–ΔΔCt^ using 3 multiple pores in each sample.

For clinical analyses, pre-treatment miR-183 values were dichotomized at 1.77, which corresponded to the cohort median and was prespecified to facilitate interpretability of efficacy and survival comparisons.

### 2.3 Statistical analysis

SPSS 24.0 software was implemented to process the data. Counting data were exhibited as *n* (%), and analyzed by χ^2^ test. Measurement data were exhibited as ($\bar{x}\pm s$), and compared by *t*-test. Kaplan-Meier curve method was implemented to draw the survival curve, and log-rank test was implemented to analyze the difference between the survival curves. Cox regression model was implemented to analyze the prognostic factors of NSCLC patients. *P* < 0.05 indicated the difference was significant.

## 3 Results

### 3.1 Therapeutic effect of EGFR-TKI and serum miR-183 expression in NSCLC patients with different therapeutic effects

**FIGURE 1:**
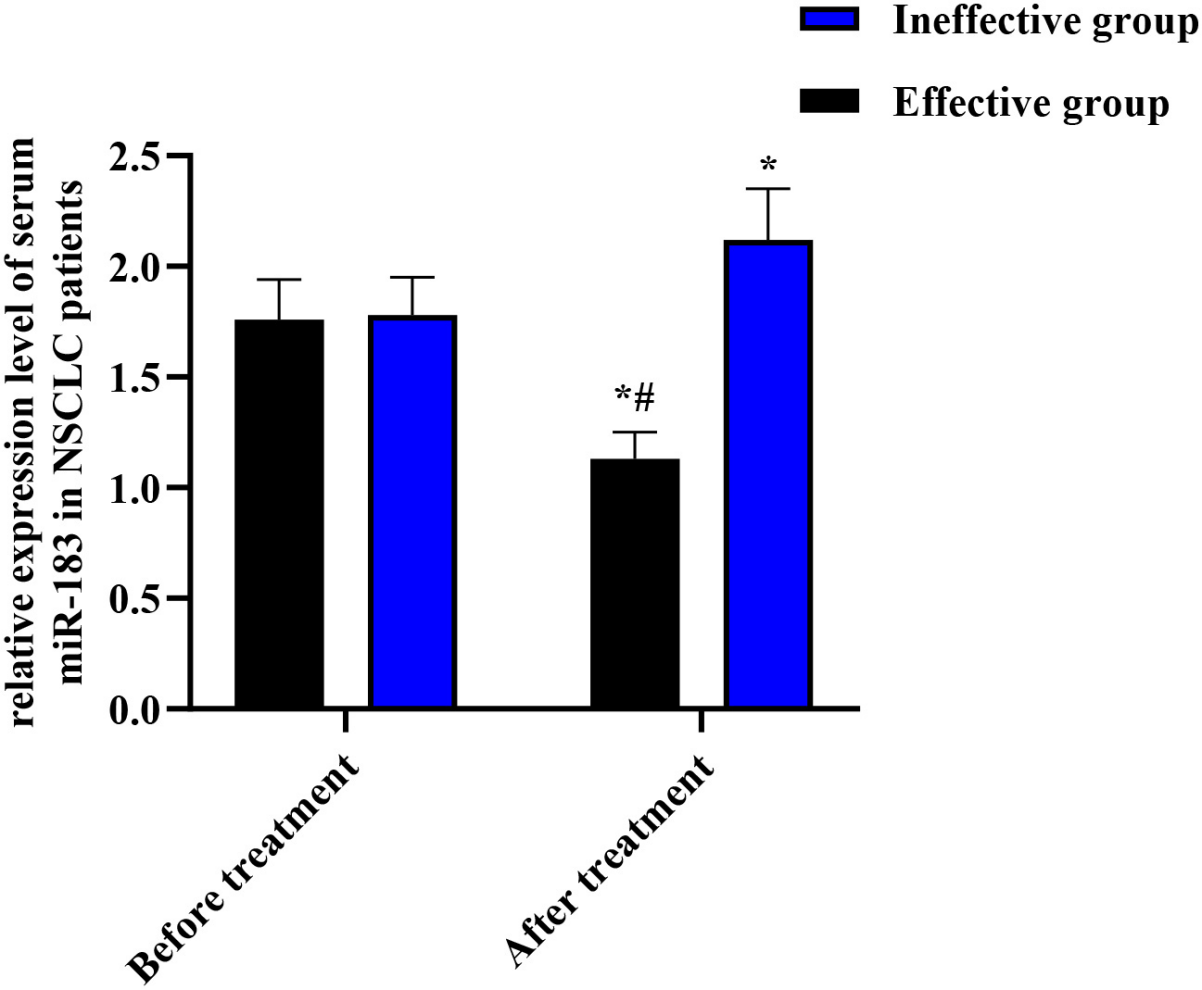
Relative expression level of serum miR-183 in NSCLC patients with different therapeutic effects. *P < 0.05, compared with before treatment. ^#^*P* < 0.05, compared with ineffective group.

After EGFR-TKI treatment, 40 of the 150 NSCLC patients were effective, 110 were ineffective, and the effective rate was 26.67% (40/150). Before treatment, serum miR-183 expression in 150 NSCLC patients was (1.77 ± 0.67), but no difference could be observed in serum miR-183 expression between effective group and ineffective group (*P* > 0.05). After treatment, serum miR-183 expression in effective group declined relative to before treatment and ineffective group (both *P* < 0.05), and serum miR-183 expression in ineffective group was elevated compared to before treatment (*P* < 0.05), as displayed in Figure 1.

### 3.2 Comparison of EGFR-TKI efficacy in NSCLC patients with different clinical characteristics

Epidermal growth factor receptor tyrosine kinase inhibitor was more effective in NSCLC patients without smoking history relative to those with smoking history (*P* < 0.05). Before treatment, serum miR-183 expression <1.77 was higher in NSCLC patients (*P* < 0.05). The age, sex along with histological type of NSCLC patients were not correlated with the efficacy of EGFR-TKI therapy (all *P* > 0.05), as displayed in Table 1.

### 3.3 Relationship between serum miR-183 expression and prognosis of NSCLC patients before treatment

**FIGURE 2:**
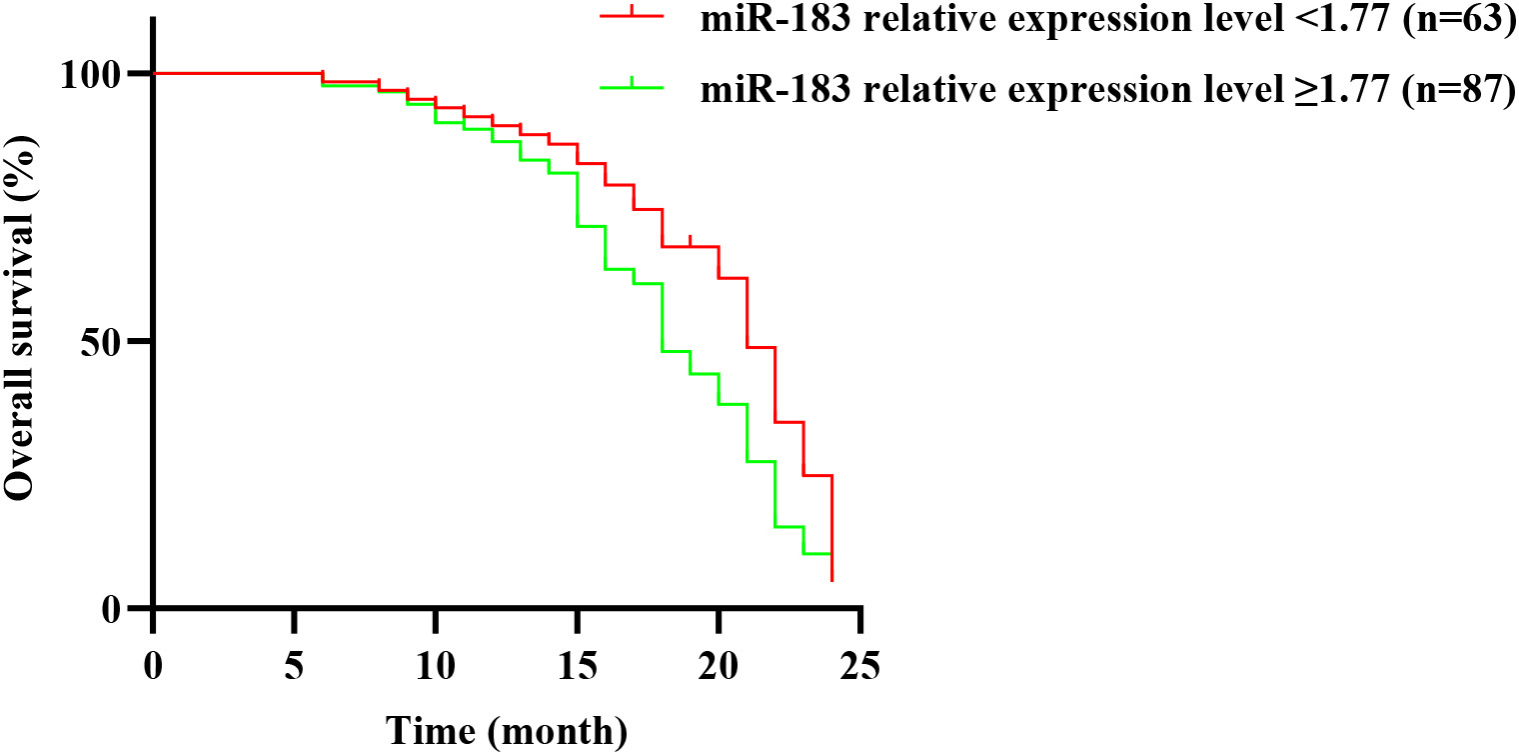
Relationship between the relative expression level of serum miR-183 and prognosis of patients with NSCLC before treatment.

All 150 NSCLC patients were followed up for 0–24 months, and 102 died, with a survival rate of 32.00% (48/150). In time-to-event analyses (Overall Survival), the median OS was 21.0 months in the low miR-183 group (<1.77) and 18.0 months in the high group (≥1.77). In a Cox proportional hazards model with miR-183 group as the sole covariate, the hazard ratio for ≥1.77 vs. <1.77 was 1.33 (95% CI, 0.90–1.96; *P* = 0.149). The log-rank test was consistent with the Cox results (χ^2^ = 2.51, *P* = 0.113) (Figure 2 and Supplementary Table 2).

### 3.4 Factors influencing survival in NSCLC patients received EGFR-TKI therapy

Multivariate Cox regression analysis was implemented with the age of NSCLC patients (<65 years = 0, ≥65 years = 1), gender (female = 0, male = 1), smoking history (no = 0, yes = 1), serum miR-183 expression before treatment (<1.77 = 0, ≥1.77 = 1), and the prognosis of NSCLC patients as the dependent variable (survival = 0, death = 1), multivariate Cox regression analysis was performed. The results manifested that serum miR-183 expression was an independent influencing factor for survival of NSCLC patients taking EGFR-TKI osimertinib (all *P* < 0.05), as displayed in Table 2.

**TABLE 2:** Cox regression analysis of factors influencing survival in NSCLC patients treated with EGFR-TKI.

Variate	*B*-value	SE value	Wald χ^2^ value	*P*-value	HR (95% CI)
Age	0.095	0.113	0.764	0.382	1.105 (0.887, 1.376)
Gender	0.076	0.104	0.508	0.475	1.073 (0.873, 1.315)
Smoking history	0.042	0.107	0.167	0.675	1.043 (0.837, 1.289)
Relative expression level of miR-183 before treatment	0.563	0.234	5.874	0.013	1.762 (1.115, 2.782)

## 4 Discussion

Non-small cell lung cancer belongs to the most frequent type of lung cancer, with high morbidity as well as mortality, poor prognosis, and serious influence on patients’ quality of life (22). Chemotherapy belongs to the main therapy for advanced NSCLC patients, but its poor efficacy and toxic side effects limit its clinical application (23). Literatures have displayed that EGFR-TKI targeted therapy has become the preferred therapy for advanced NSCLC patients possessing EGFR gene sensitive mutations (24). The outcomes of this research demonstrated that the effective rate of EGFR-TKI in the treatment of NSCLC patients was 26.67%, suggesting that EGFR-TKI has certain clinical efficacy in the therapy of advanced NSCLC (25).

Clinical imaging methods are often used to assess the therapeutic effect of NSCLC patients, but imaging examinations are expensive and have radiation side effects, which are not suitable for repeated examination in a short time, and there are certain limitations (26). The serum markers have a certain value in evaluating the therapeutic effect of malignant tumors, and the serological indicators have the characteristics of convenient sampling, rapid detection, and good repeatability (27). MiRNAs are widely distributed in peripheral blood and various organs and tissues. They can regulate biological processes containing cell proliferation, apoptosis, and inflammatory response, and have a crucial regulatory function in the development of various cancers, NSCLC included (28, 29). As reported previously, miR-183-5p, a part of the miR-183 family, is dysregulated in NSCLC acts as an oncogenic miRNA that can accelerate the tumor growth as well as metastasis of NSCLC via targeting PTEN (18). MiR-183 promotes growth of NSCLC cells by suppressing FoxO1 (19). High expression of serum miR-183 family is linked to overall poor survival in lung cancer patients (15). The above studies indicate that the abnormal expression of miR-183 is strongly linked to the progression of NSCLC.

The outcomes of this research exhibited that after EGFR-TKI therapy, serum miR-183 expression in the effective group was lower relative to that before treatment together with the ineffective group, and serum miR-183 expression in the ineffective group was higher relative to that before treatment. It is suggested that miR-183 may have a crucial important impact on the development of NSCLC, and the serum miR-183 level before treatment is related to the therapeutic effect of EGFR-TKI in advanced NSCLC patients. It is speculated that miR-183 may affect signal transduction through binding with target genes, thus affecting biological processes containing proliferation, apoptosis, along with invasion of NSCLC cells, and thus affecting the development of NSCLC (30). In particular, miR-183 has been shown to directly target PTEN and FOXO1 – both are key negative regulators of the EGFR signaling pathway/PI3K/AKT pathway. Specifically, in NSCLC, overexpression of miR-183-5p suppresses PTEN expression, leading to activation of AKT signaling and promotion of tumor growth and metastasis (18). Moreover, miR-183 suppresses FOXO1 translation by binding its 3’-UTR, which enhances NSCLC cell proliferation *in vitro* and tumor growth *in vivo* (19). These interactions raise the possibility that miR-183 might modulate response to EGFR-TKI therapy via off-target effects on PTEN/FOXO1 and downstream signaling. At the same time, EGFR-TKI can compete with adenosine triphosphate for tyrosine kinase sites, thereby inhibiting intracellular tyrosine kinase phosphorylation, epidermal growth factor receptor signaling, and cancer cell proliferation, growth, and angiogenesis, thus playing an anti-tumor role in progressive and recurrent NSCLC (31). Therefore, miR-183 may be the target of EGFR-TKI. The level of miR-183 changed after EGFR-TKI therapy in NSCLC patients, suggesting that the change of miR-183 level could reflect the therapeutic effect of EGFR-TKI. In addition, followed by EGFR-TKI therapy, the lesions of NSCLC patients in the effective group were significantly reduced, the growth and metastasis of malignant tumors were inhibited, and the relative expression of serum miR-183 might be affected.

The outcomes of this study revealed that patients with no smoking history and serum miR-183 relative expression level < 1.77 before treatment had a better effect on EGFR-TKI treatment. The survival rate of NSCLC patients with serum miR-183 expression level < 1.77 after EGFR-TKI treatment was higher than those with serum miR-183 expression level ≥ 1.77 before treatment. Multivariate Cox regression analysis presented that serum miR-183 relative expression level before treatment was an independent factor influencing the survival of NSCLC patients taking EGFR-TKI. This suggests that abnormal expression level of serum miR-183 before treatment may affect the prognosis of NSCLC patients after EGFR-TKI treatment. Dynamic monitoring of serum miR-183 level is conducive to evaluating the prognosis of patients.

To our knowledge, few studies have specifically linked baseline circulating miR-183 to subsequent EGFR-TKI efficacy in advanced NSCLC; our data address this gap and provide impetus for prospective validation. However, this single-center study has modest sample size, which limits the precision and generalizability of the findings. We therefore emphasize estimation with confidence intervals and interpret results as hypothesis-generating. To mitigate overfitting, we restricted model complexity to a small, clinically justified covariate set. Future prospective, multi-center studies with larger and more diverse cohorts are needed to confirm these observations and to refine clinical applicability.

## 5 Conclusion

Low serum miR-183 expression level before treatment is closely linked to the efficacy and prognosis of advanced NSCLC patients who received EGFR-TKI therapy. Monitoring serum miR-183 level may be helpful to evaluate the prognosis of patients with NSCLC. Nevertheless, the sample size of our research is small, and the experimental findings may be biased. The sample size will be increased for further exploration in the future.

## Data Availability

The original contributions presented in this study are included in this article/Supplementary material, further inquiries can be directed to the corresponding author.
