# Supplementary material for: Evaluation of the efficacy as well as prognosis of targeted therapy for advanced non-small cell lung cancer patients with different expression of miR-183 family in body fluids
Source: Front Med (Lausanne). 2026 Jan 12;12:1738202. doi: 10.3389/fmed.2025.1738202 (PMC12833209; doi:10.3389/fmed.2025.1738202)
Supplement: Supplementary file 1 [file Table_1.docx]

Supplementary Table 1 Eligibility criteria

| Inclusion criteria | Exclusion criteria |
| --- | --- |
| 1) Advanced NSCLC diagnosed as adenocarcinoma. 2) Complete baseline clinical data and serum sample available. 3) Received EGFR‑TKI treatment. 4) Provided written informed consent. | 1) Severe cardiovascular disease or other serious comorbidities. 2) Lost to follow‑up. 3) Unclear pathological diagnosis. 4) Incomplete clinical information. |
