# Supplementary material for: Evaluation of the efficacy as well as prognosis of targeted therapy for advanced non-small cell lung cancer patients with different expression of miR-183 family in body fluids
Source: Front Med (Lausanne). 2026 Jan 12;12:1738202. doi: 10.3389/fmed.2025.1738202 (PMC12833209; doi:10.3389/fmed.2025.1738202)
Supplement: Supplementary file 2 [file Table_2.docx]

Supplementary Table S2. Cox model and survival estimates

| Model ID | Outcome | Predictor | HR | 95% CI lower | 95% CI upper | P (Wald) | Log-rank χ² | Log-rank P | N (low / high) | Events (low / high) | Median OS (low / high) |
| --- | --- | --- | --- | --- | --- | --- | --- | --- | --- | --- | --- |
| Primary Cox | Overall survival (months) | miR-183 ≥1.77 vs <1.77 | 1.331 | 0.903 | 1.962 | 0.1488 | 2.513 | 0.1129 | 87 / 63 | 51 / 51 | 21.0 / 18.0 |
